# Evolution of Influenza A Virus H7 and N9 Subtypes, Eastern Asia

**DOI:** 10.3201/eid1910.130609

**Published:** 2013-10

**Authors:** Camille Lebarbenchon, Justin D. Brown, David E. Stallknecht

**Affiliations:** Centre de Recherche et de Veille sur les Maladies Émergentes dans l’Océan Indien, Sainte Clotilde, Reunion (C. Lebarbenchon);; Université de la Réunion, Saint-Denis, Reunion (C. Lebarbenchon);; College of Veterinary Medicine, University of Georgia, Athens, Georgia, USA (J.D. Brown, D.E. Stallknecht)

**Keywords:** influenza, avian influenza virus, influenza A virus, viruses, hemagglutinin, neuraminidase, H7 subtype, N9 subtype, wild birds, evolution, gene flow, live-bird markets, poultry, eastern Asia

## Abstract

Influenza A viruses are a threat to poultry and human health. We investigated evolution of influenza A virus H7 and N9 subtypes in wild and domestic birds. Influenza A(H7N9) virus probably emerged after a long silent circulation in live poultry markets in eastern Asia.

Emergence of influenza A(H7N9) virus in China raised concerns about potential virus adaptation to mammals and human-to-human transmission (*1**,**2*). Investigations of virus sources and vectors are needed because they will provide useful information about influenza A(H7N9) virus subtype evolution and adaptation processes. Wild waterbirds are natural hosts for influenza A viruses and are sources for introduction of virus into poultry, in which the viruses adapt and sometimes evolve toward increased virulence (H5 and H7 virus subtypes). Although H7 subtype influenza A viruses have been isolated from wild birds worldwide, the role of these hosts in emergence, maintenance, and potential intercontinental spread of influenza A(H7N9) virus has not been determined.

We analyzed molecular evolution of H7 (hemagglutinin) and N9 (neuraminidase) subtypes of avian influenza virus. The purpose of this study was to investigate the recent evolutionary history of H7 and N9 virus subtypes in eastern Asia and identify the most recent wild bird ancestor of influenza A(H7N9) virus hemagglutinin and neuraminidase.

## The Study

To assess global phylogeny of influenza A virus H7 and N9 subtypes, we analyzed 715 hemagglutinin and 309 neuraminidase nucleotide sequences of viruses isolated during 1927–2012 worldwide (Technical Appendix). Bayesian Markov Chain Monte Carlo coalescent analyses were conducted to investigate recent evolutionary history of influenza A virus H7 and N9 subtypes in eastern Asia by using BEAST version 1.7.4 (*3**,**4*).

**Figure 1:**
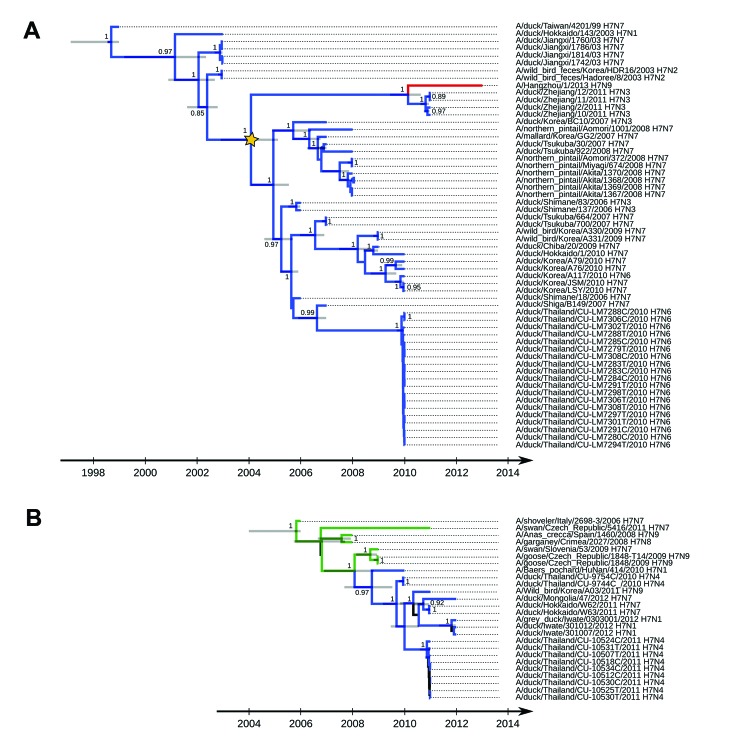
Maximum clade credibility trees for co-circulating influenza A virus H7 subtype genetic lineages, eastern Asia. A) Clade A. B) Clade B. Values along the branches are posterior probability values >0.8. Gray bars indicate 95% highest posterior density for times of the most recent common ancestors; blue indicates viruses isolated in Asia; green indicates viruses isolated in Europe (details on locations and associated posterior probabilities are shown in the online Technical Appendix, wwwnc.cdc.gov/EID/article/19/10/13-0609-Techapp1.pdf); red indicates A/Hangzhou/1/2013(H7N9) virus; and yellow star indicates most recent common ancestral influenza virus among A/Hangzhou/1/2013(H7N9) virus, Zhejiang domestic ducks viruses (H7N3), and influenza A virus H7 subtype circulating in wild birds.

Phylogenetic analyses showed maintenance of influenza A virus H7 subtypes in wild birds in eastern Asia since 1999 (clade A) (Figure 1, panel A). More specifically, circulation has been restricted mainly to the eastern Asia flyway; most viruses isolated from wild birds were from Japan and South Korea. This local perpetuation in wild birds has favored several independent introductions of viruses into poultry in Japan, South Korea, eastern China (Jiangxi and Zhejiang Provinces), and Thailand. A genetically different virus H7 subtype lineage was detected in Europe and Asia during 2006–2012 (clade B) (Figure 1, panel B). These results suggest that ≥2 influenza A virus H7 subtypes co-circulated in eastern Asia during that period. In Japan, replacement of influenza A virus H7 subtypes that were circulating in wild ducks during 2008 (clade A) may have occurred because recent viruses isolated from ducks (2011–2012) all belong to clade B. The same pattern was observed in Thailand: influenza A virus H7 subtypes isolated in 2011 were genetically different from most viruses isolated in 2010.

This pattern suggests that the old genetic lineage of influenza A virus H7 subtypes that circulated in eastern Asia since 1999 (clade A) may have been progressively replaced by a more recent lineage (clade B). Clustering of clade B viruses with those isolated in Europe suggest that gene flow has recently occurred in Eurasia, probably as the result of waterfowl migrations and poultry trade. However, the European origin of viruses from Asia was not supported on the basis of phylogeographic analysis (Technical Appendix).

Consistent with results of previous studies (*1**,**2**,**5**,**6*), our results indicate that hemagglutinin of human influenza A(H7N9) viruses belongs to clade A and is most genetically related to influenza viruses isolated from domestic ducks at live-poultry markets in Zhejiang Province, China (*7*). Our findings further indicate that hemagglutinin of human influenza virus did not evolve from the H7 HA circulating in these domestic birds but was derived from a common ancestral influenza A virus circulating in an unidentified host during 2010. The most recent common ancestral influenza A virus among A/Hangzhou/1/2013(H7N9) virus, Zhejiang domestic duck(H7N3) virus, and influenza A virus H7 subtype circulating in wild birds could be dated to 2004 (Figure 1, panel A), indicating that silent introduction and circulation of influenza A virus H7 subtypes in domestic animals might have occurred in this virus before influenza A(H7N9) virus was identified in humans (*8*).

Limited epidemiologic and genetic information about influenza A virus H7 subtype circulating in eastern China during 2004–2011 precludes more precise conclusions on origins of human influenza A(H7N9) virus and relatedness to influenza A virus circulating in wild birds. However, on the basis of genetic analyses of recently isolated viruses from chickens, pigeons, and the environment, maintenance and genetic reassortment of emerging influenza A(H7N9) virus might have occurred in live poultry markets in Shanghai, China (*5**,**7*).

**Figure 2:**
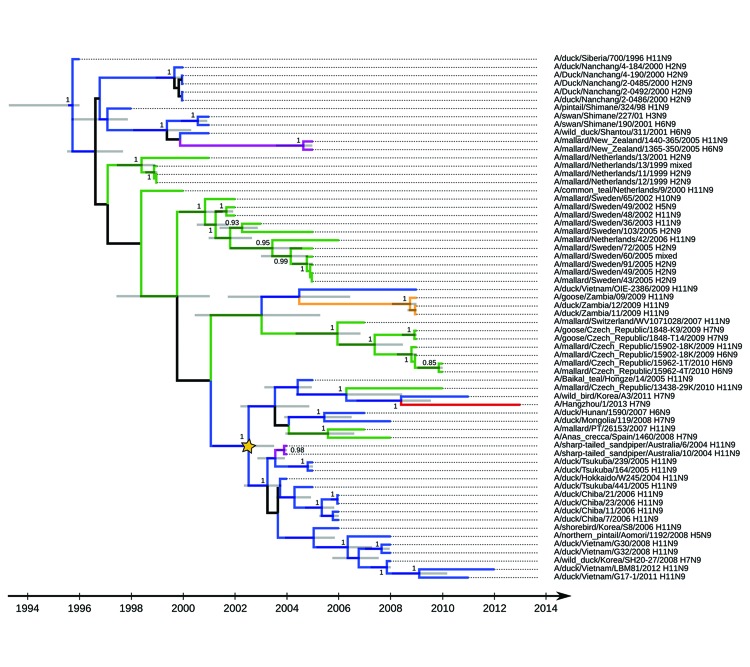
Maximum clade credibility tree for influenza A virus N9 subtype genetic lineages in Eurasia. Values along branches are posterior probability values >0.8. Gray bars indicate the 95% highest posterior density for times of the most recent common ancestors. Blue indicates viruses isolated in Asia; green indicates viruses isolated in Europe; purple indicates viruses isolated in Oceania; orange indicates viruses isolated in Africa (details on locations and associated posterior probabilities are shown in the online Technical Appendix, wwwnc.cdc.gov/EID/article/19/10/13-0609-Techapp1.pdf); red indicates A/Hangzhou/1/2013(H7N9) virus; and yellow star indicates the basis of influenza A(H11N9) virus genetic lineage from Asia.

The phylogenetic structure we observed for influenza A virus subtype N9 suggests that gene flow has occurred since 1996 among Europe, Africa, Asia, and Oceania (Figure 2; Technical Appendix). Analyses showed circulation of influenza A virus subtype N9 (mainly H11N9 subtype) (Figure 2) in eastern Asia since 2003, and evidence of virus dispersal to Europe and Australia and reassortments with hemagglutinin of avian influenza virus subtypes H5, H6, and H7. Consistent with results of other studies (*1**,**2**,**5**,**6*), we found that neuraminidase of A/Hangzhou/1/2013(H7N9) virus was closely related to that of A/wild bird/Korea/A3/2011(H7N9) virus (*9*). However, our estimate of the time of the most recent common ancestral influenza A virus between these 2 viruses was earlier (2008) than suggested (*6*).

## Conclusions

Our findings suggest that neuraminidase of human influenza A(H7N9) virus might have originated from influenza A(H11N9) viruses that circulated in eastern China, although limited information about influenza A virus N9 subtypes circulating in wild birds in this region represents a major challenge to identifying the donor of influenza A(H7N9) virus neuraminidase. Reassortments between influenza A(H11N9) viruses from Asia and influenza A(H1N3 and H7N3) viruses circulating in live-poultry markets in Zhejiang Province were documented in domestic duck in 2011 in nearby Jiangsu Province (A/duck/Jiangsu/10-d4/2011(H1N3) (*10*) In a similar fashion, influenza A(H7N9) virus could have resulted from silent circulation and reassortment between influenza (H7N3 and H11N9) viruses in live-poultry markets in the Shanghai region.

As in a study on influenza A virus H7 subtype evolution in wild birds and poultry (*11*), we found no evidence of spillover of influenza A virus H7 subtype from domestic to wild birds and subsequent long-term maintenance in eastern Asia. Although we cannot formally exclude that local transmission of influenza A virus from domestic to wild birds has occurred, lack of evidence for reintroduction of poultry-adapted viruses into wild birds suggests there has been little to no dissemination of influenza A(H7N9) virus by waterfowl along their migratory flyways. Increasing adaptation of this virus to mammals (*2*) is unlikely to favor spillover and spread by migratory birds. However, development and maintenance of influenza A virus surveillance programs for wild waterfowls worldwide are needed to confirm this possibility (*12**,**13*).

In eastern Asia, 2 major influenza A virus H7 subtype genetic lineages have recently circulated in wild and domestic birds, and there has been potential replacement of the older lineage (clade A). Hemagglutinin of influenza A(H7N9) virus belongs to clade A. Genetic data indicate that the most recent ancestral wild bird–origin virus for A/Hangzhou/1/2013(H7N9) virus and Zhejiang domestic duck viruses can be dated to 2004. The influenza A(H11N9) virus that circulated in eastern Asia for ≈10 years, with associated intercontinental gene flows and reassortments, may be the donor of influenza A(H7N9) virus neuraminidase. Hosts and areas in which ancestral viruses have been maintained are unknown. However, influenza A(H7N9) virus probably emerged after a long silent circulation in live poultry markets in eastern Asia.

Technical Appendix Global phylogeny of influenza A virus H7 and N9 subtypes and phylogeography of recent H7 and N9 subtype genetic lineages in eastern Asia.

## References

[1] Gao R, Cao B, Hu Y, Feng Z, Wang D, Hu W, Human infection with a novel avian-origin influenza A (H7N9) virus. N Engl J Med. 2013;368:1888–97. 10.1056/NEJMoa1304459 23577628

[2] Kageyama T, Fujisaki S, Takashita E, Xu H, Yamada S, Uchida Y, Genetic analysis of novel avian A(H7N9) influenza viruses isolated from patients in China, February to April 2013. Euro Surveill. 2013;18:20453 . 23594575 PMC6296756

[3] Drummond AJ, Rambaut A. BEAST: Bayesian evolutionary analysis by sampling trees. BMC Evol Biol. 2007;7:214. 10.1186/1471-2148-7-214 17996036 PMC2247476

[4] Lemey P, Rambaut A, Drummond AJ, Suchard MA. Bayesian phylogeography finds its roots. PLOS Comput Biol. 2009;5:e1000520. 10.1371/journal.pcbi.1000520 19779555 PMC2740835

[5] Shi JZ, Deng GH, Liu PH, Zhou JP, Guan LZ, Li WH, Isolation and characterization of H7N9 viruses from live poultry markets: implication of the source of current H7N9 infection in humans. Chin Sci Bull. 2013;58:1857–63. 10.1007/s11434-013-5873-4

[6] Liu D, Shi W, Shi Y, Wang D, Xiao H, Li W, Origin and diversity of novel avian influenza A H7N9 viruses causing human infection: phylogenetic, structural and coalescent analyses. Lancet. 2013;381:1926–32. 10.1016/S0140-6736(13)60938-1 23643111

[7] Hai-bo W, Ru-feng L, En-kang W, Jin-biao Y, Yi-ting W, Qiao-gang W, Sequence and phylogenetic analysis of H7N3 avian influenza viruses isolated from poultry in China in 2011. Arch Virol. 2012;157:2017–21. 10.1007/s00705-012-1370-3 22752840

[8] Jonges M, Meijer A, Fouchier RA, Koch G, Li J, Pan JC, Guiding outbreak management by the use of influenza A(H7Nx) virus sequence analysis. Euro Surveill. 2013;18:20460 . 23611030

[9] Kim HR, Park CK, Lee YJ, Oem JK, Kang HM, Choi JG, Low pathogenic H7 subtype avian influenza viruses isolated from domestic ducks in South Korea and the close association with isolates of wild birds. J Gen Virol. 2012;93:1278–87. 10.1099/vir.0.041269-0 22422062

[10] Chen C, Zhao G, Gu X, Gu M, Hu J, Li Q, Complete genomic sequence of a novel reassortant H11N3 influenza virus isolated from domestic ducks in Jiangsu, China. J Virol. 2012;86:11950–1. 10.1128/JVI.02167-12 23043179 PMC3486340

[11] Lebarbenchon C, Stallknecht DE. Host shifts and molecular evolution of H7 avian influenza virus hemagglutinin. Virol J. 2011;8:328. 10.1186/1743-422X-8-328 21711553 PMC3141685

[12] Munster VJ, Wallensten A, Baas C, Rimmelzwaan GF, Schutten M, Olsen B, Mallards and highly pathogenic avian influenza ancestral viruses, northern Europe. Emerg Infect Dis. 2005;11:1545–51. 10.3201/eid1110.050546 16318694 PMC3366752

[13] Lebarbenchon C, Feare CJ, Renaud F, Thomas F, Gauthier-Clerc M. Persistence of highly pathogenic avian influenza viruses in natural ecosystems. Emerg Infect Dis. 2010;16:1057–62 . 10.3201/eid1607.090389 20587174 PMC3321889

