# Supplementary material for: Evolution of Influenza A Virus H7 and N9 Subtypes, Eastern Asia
Source: Emerg Infect Dis. 2013 Oct;19(10):1635–8. doi: 10.3201/eid1910.130609 (PMC3810757; doi:10.3201/eid1910.130609)
Supplement: Technical Appendix — Global phylogeny of influenza A virus H7 and N9 subtypes and phylogeography of recent H7 and N9 subtype genetic lineages in eastern Asia. [file 13-0609-Techapp-s1.pdf]

Article DOI: http://dx.doi.org/10.3201/eid1910.130609

Evolution of Influenza A Virus H7 and N9 Subtypes, Eastern Asia

Technical Appendix

Global Phylogeny of Influenza A Virus H7 and N9 Subtypes

A total of 714 hemagglutinin and 308 neuraminidase complete nucleotide sequences of influenza A viruses isolated in wild and domestic birds were downloaded from the Influenza Sequence Database (*1*) on April 15, 2013. The hemagglutinin and neuraminidase sequences of the A/Hangzhou/1/2013 (H7N9) virus human isolate were also included. Sequences with unidentified hosts, duplicate sequences from the same strain, and those identified as reflecting potential laboratory errors were excluded from the dataset (*2*,*3*). The coding region of nucleotide sequences was aligned by using the CLC Sequence Viewer version 6.6.2 (CLC Bio, Aarhus, Denmark) (sequence alignments are available from the authors). A maximum-likelihood analysis was performed by using R 2.14.1 software (www.R-project.org) and Phangorn version 1.6.4 (*4*) with the general time reversible evolutionary model, an estimation of the proportion of invariable sites, and the nucleotide heterogeneity of substitution rates. Nodal supports were assessed with 1,000 bootstrap replicates. Phylogenetic trees are shown in Technical Appendix Figures 1, 2.

Phylogeography of Recent Influenza A Virus H7 and N9 Subtype Genetic Lineages in Eastern Asia

Bayesian Markov Chain Monte Carlo coalescent analyses were performed to investigate the recent evolutionary history of influenza A virus H7 and N9 subtypes in eastern Asia. Location states and associated posterior probabilities for internal nodes were obtained by using the program BEAST 1.7.4 (*5*) according to a described method (*6*). The uncorrelated exponential molecular clock was selected according to Bayes factors comparison with estimates obtained for strict and uncorrelated lognormal local clocks. The SRD06 nucleotide substitution model (*7*) and a Bayesian skyline coalescent tree prior were used in all simulations (*8*). Analyses were

performed with a chain length of 30–60 million generations sampled every 1,000 iterations; the first 10% of trees were discarded as burn-in. Complementary information for results derived from these analyses is shown in Technical Appendix Figures 3, 4.

References

1. Bao Y, Bolotov P, Dernovoy D, Kiryutin B, Zaslavsky L, Tatusova T, et al. The influenza virus resource at the National Center for Biotechnology Information. J Virol. 2008;82:596–601. PubMed http://dx.doi.org/10.1128/JVI.02005-07

2. Krasnitz M, Levine AJ, Rabadan R. Anomalies in the influenza virus genome database: new biology or laboratory errors? J Virol. 2008;82:8947–50. PubMed http://dx.doi.org/10.1128/JVI.00101-08

3. Lebarbenchon C, Stallknecht DE. Host shifts and molecular evolution of H7 avian influenza virus hemagglutinin. Virol J. 2011;8:328. PubMed http://dx.doi.org/10.1186/1743-422X-8-328

4. Schliep KP. Phangorn: phylogenetic analysis in R. Bioinformatics. 2011;27:592–3. PubMed http://dx.doi.org/10.1093/bioinformatics/btq706

5. Drummond AJ, Rambaut A. BEAST: Bayesian evolutionary analysis by sampling trees. BMC Evol Biol. 2007;7:214. PubMed http://dx.doi.org/10.1186/1471-2148-7-214

6. Lemey P, Rambaut A, Drummond AJ, Suchard MA. Bayesian phylogeography finds its roots. PLOS Comput Biol. 2009;5:e1000520. PubMed http://dx.doi.org/10.1371/journal.pcbi.1000520

7. Shapiro B, Rambaut A, Drummond AJ. Choosing appropriate substitution models for the phylogenetic analysis of protein-coding sequences. Mol Biol Evol. 2006;23:7–9. PubMed http://dx.doi.org/10.1093/molbev/msj021

8. Drummond AJ, Rambaut A, Shapiro B, Pybus OG. Bayesian coalescent inference of past population dynamics from molecular sequences. Mol Biol Evol. 2005;22:1185–92. PubMed http://dx.doi.org/10.1093/molbev/msi103

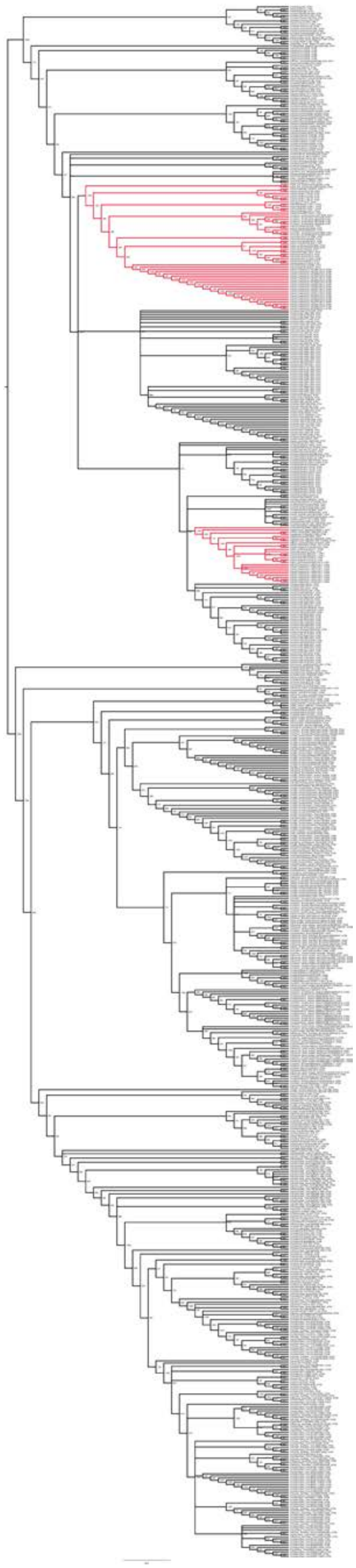

Technical Appendix Figure 1 (click figure to enlarge). Maximum likelihood consensus tree derived from 715 influenza A virus H7 subtype hemagglutinin nucleotide sequences. Red branches indicate recent genetic lineages of H7 subtype viruses that circulated in eastern Asia, and for which detailed evolutionary history was investigated by using coalescent analyses. Scale bar indicates nucleotide substitutions per site.

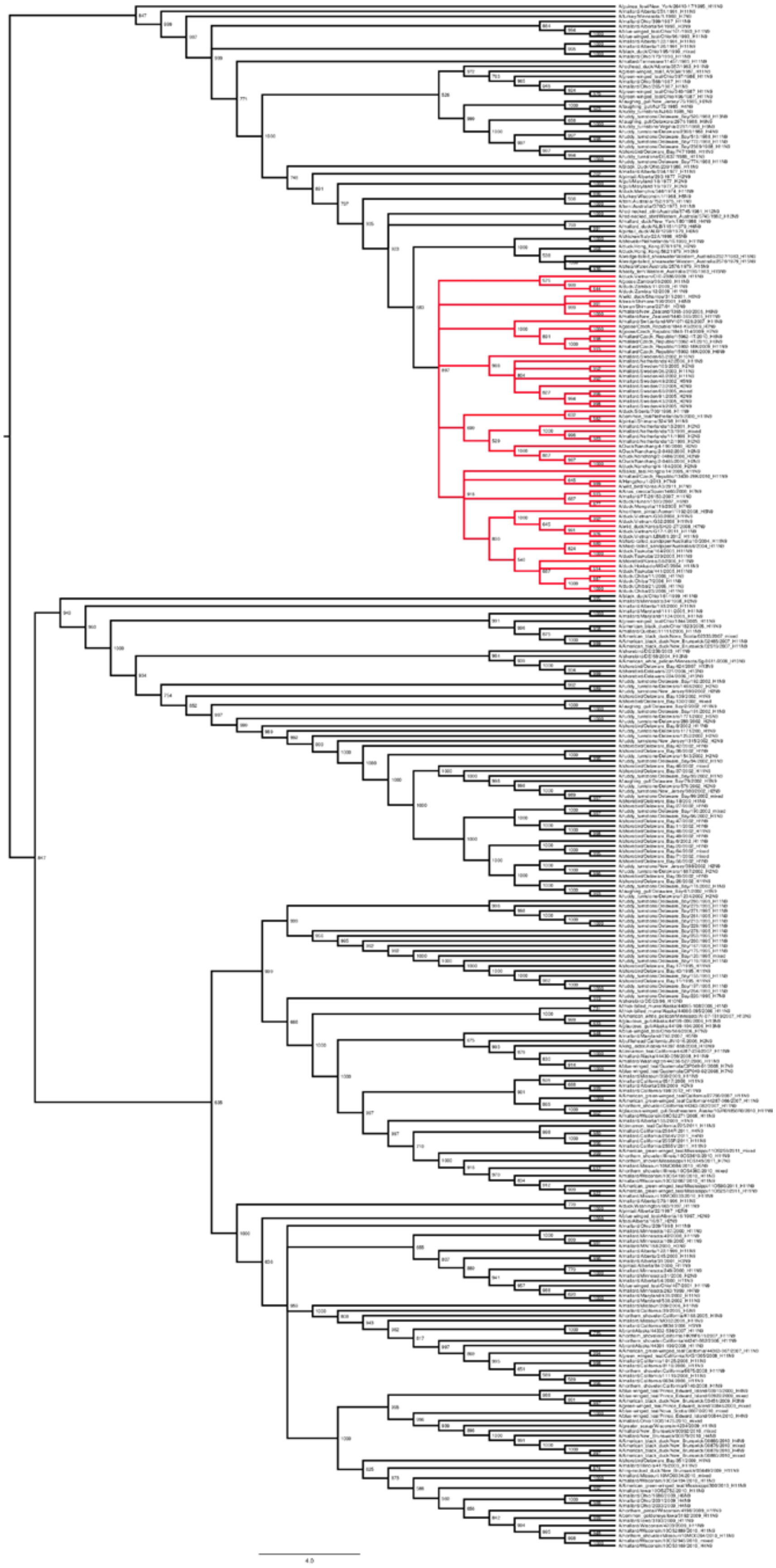

Technical Appendix Figure 2 (click figure to enlarge). Maximum-likelihood consensus tree derived from 309 influenza A virus N9 subtype neuraminidase nucleotide sequences. Red branches indicate the Eurasian lineage of N9 subtype viruses for which detailed evolutionary history was investigated with coalescent analyses. Scale bar indicates nucleotide substitutions per site.

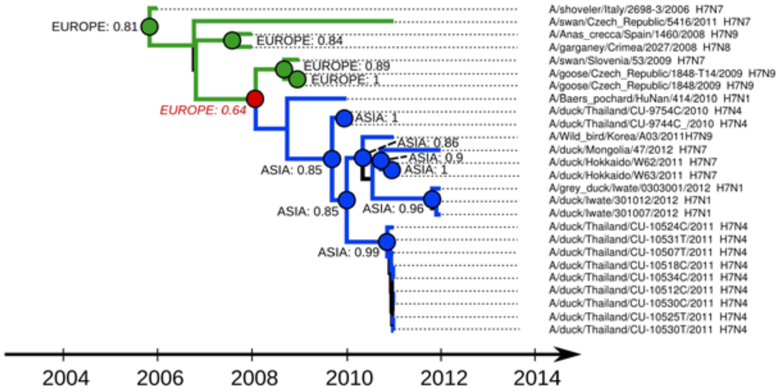

Technical Appendix Figure 3. Maximum clade credibility tree for clade B of influenza A virus H7 hemagglutinin subtypes. Branches are colored according to most probable location, as obtained by coalescent analysis. Green indicates Europe and blue indicates Asia. For internal nodes, locations and associated posterior probabilities are reported when >0.8. Supporting value for the European ancestor of the Asian lineage (red dot) is indicated in italics.

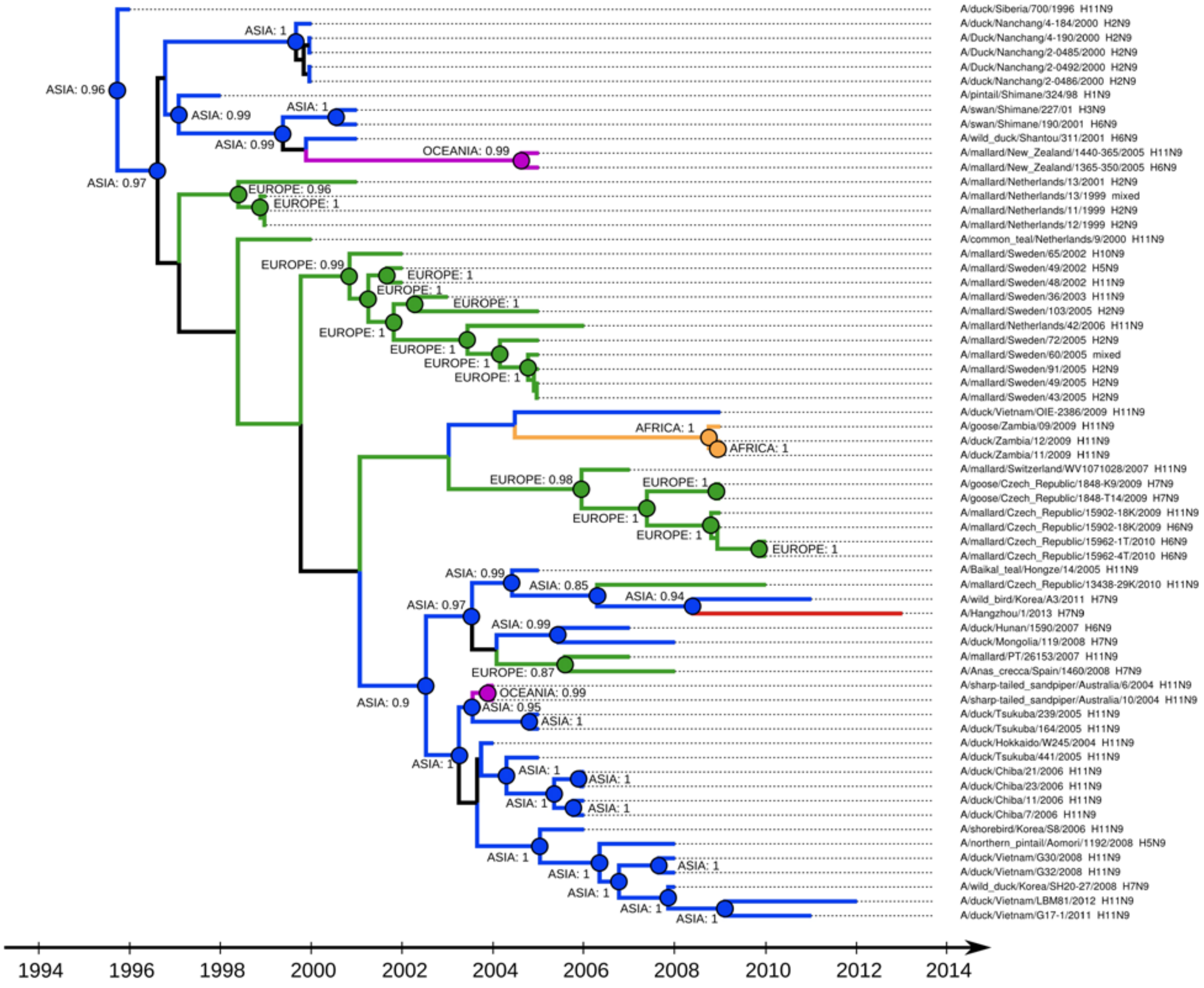

Technical Appendix Figure 4. Maximum clade credibility tree for influenza A virus N9 neuraminidase subtypes recently circulating in Eurasia. Branches are colored according to most probable location, as obtained by coalescent analysis. Blue indicates Asia; purple indicates Oceania; green indicates Europe; orange indicates Africa. For internal nodes, locations and associated posterior probabilities are reported when >0.8. Red indicates A/Hangzhou/1/2013 (H7N9) virus.
